# The organization of microtubules and Tau in oligodendrocytes: Tau pathology in damaged oligodendrocytes

**DOI:** 10.3389/fcell.2022.950682

**Published:** 2022-10-05

**Authors:** Tomohiro Torii, Tomohiro Miyasaka, Hiroaki Misonou

**Affiliations:** ^1^ Laboratory of Ion Channel Pathophysiology, Graduate School of Brain Science, Doshisha University, Kyoto, Japan; ^2^ Center for Research in Neurodegenerative Diseases, Doshisha University, Kyoto, Japan; ^3^ Department of Neuropathology, Faculty of Life and Medical Sciences, Doshisha University, Kyoto, Japan

**Keywords:** tau, myelin, oligodendrocyte, myelination, demyelination

## Abstract

Tau is abundantly expressed in neurons, however previous reports and our recent study showed tau also exist in oligodendrocytes. Also the expression levels of tau are dramatical changed in hypomyelination model rat and in demyelination region of stroke model mice. The review demonstrate microtubule and its binding partner Tau might be necessary for oligodendrocyte function based on previous reports.

Microtubules and the binding partners essentially control cellular functions during cell proliferation, differentiation, and migration in mammalian cells (Brouhard and Rice, 2018). Microtubules are polymers of alpha-tubulin/beta-tubulin subunits, and the stability and/or instability of microtubules (microtubule dynamics) are controlled by several microtubule-associated protein family proteins.

Among them, Tau is expressed mainly in neurons in the brain and has been used as a neuronal (and axonal) marker. However, its expression in oligodendrocytes has also been implicated (LoPresti et al., 1995; Bonetto et al., 2021). Recent studies unambiguously showed that tau is expressed in olig2-positive oligodendrocytes of mouse brain (Kubo et al., 2019) (Figure 1B) and in myelin basic protein (MBP)-positive cells of adult rat brain (Kanaan and Grabinski, 2020) using well-validated antibodies. These studies also confirmed that Tau does not present in NG2-positive oligodendrocyte precursor cells (OPCs), astrocytes, and microglia. Based on these findings, Tau is recognized to exists in mature oligodendrocytes as well as in neurons of central nervous system (CNS) (Figure 1). However, the physiological roles of Tau in oligodendrocytes is not well-understood, as the normal myelination observed in Tau conventional knockout mice (Takei et al., 2000) implicates that Tau is not indispensable for OPC migration, differentiation, and myelination.

**FIGURE 1:**
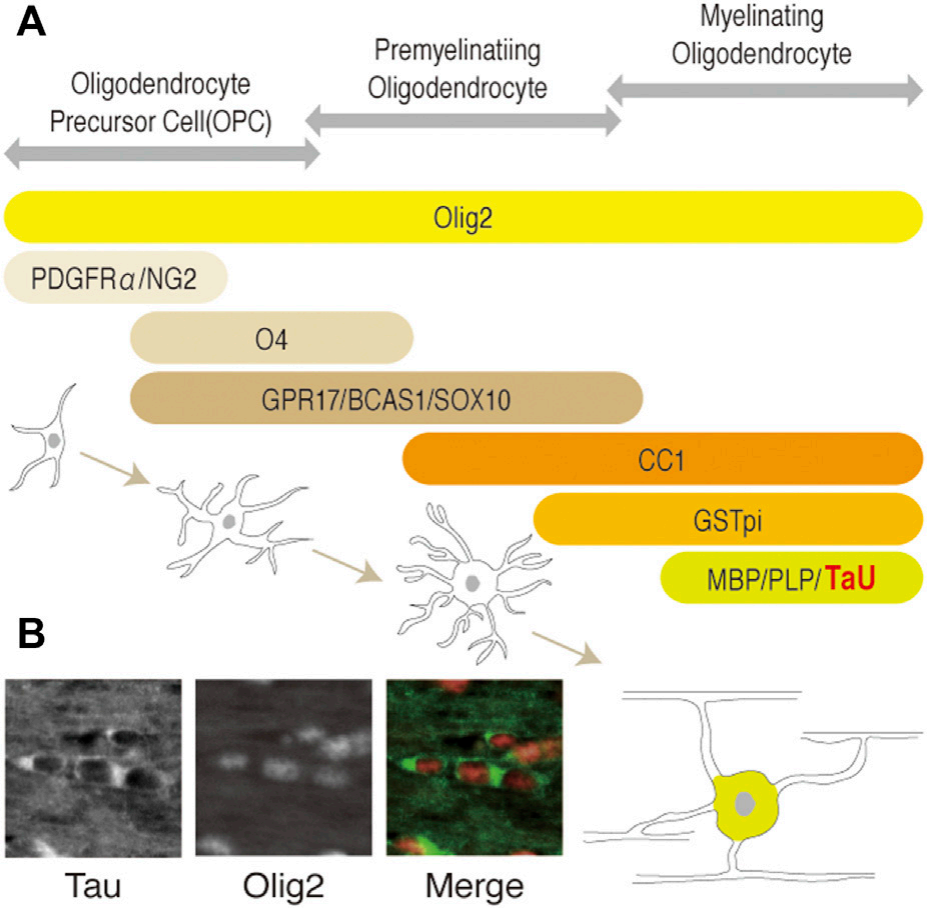
**(A)** Schematic representation of the developmental stages and stage-specific markers of the oligodendrocytes lineage. OPC, pre-myelinating oligodendrocytes, or myelinating oligodendrocytes (mature oligodendrocytes) are identified by each marker as shown. Olig2 is expressed in all cells of the lineage during development. O4, GPR17, BCAS1, and SOX10 (Ulloa-Navas et al., 2021) are pre-myelinating oligodendrocyte markers. Platelet-derived growth factorα: PDGFRα; neuron-glial antigen 2: NG2; Oligodendrocyte marker O4: O4; G-protein coupled receptor 17: GPR17; breast carcinoma amplified sequence 1: BCAS1; myelin basic protein: MBP; proteolipid protein 1: PLP1; kallikrein related peptidase: Klk6. **(B)** Representation of Tau and olig2 distribution in oligodendrocytes of adult mice brain. Cytoplasmic localization of Tau in mature oligodendrocytes of corpus callosum are shown, respectively.

In contrast, insights into the pathological roles of Tau in oligodendrocytes have emerged from a number of studies using human patient postmortem tissues and animal models. Aggregation of the phosphorylated tau, that is detected with the anti-AT8 antibody, is observed in astrocytes as well as in oligodendrocytes in the brains of patients with globular glial tauopathy (GGT) (Ahmed et al., 2013). Also, rats harboring mutation in the Tubulin beta 4a gene (Tubb4a), which exhibit hypo- and/or de-myelination, also show elevated Tau expression in oligodendrocytes (in culture) (Song et al., 1999). The Tubb4a mutation has been shown to affect microtubule dynamics such as its elongation, length, duration, and the frequency of them in oligodendrocytes of dystonia or hypomyelination with atrophy of the basal ganglia and cerebellum (H-ABC) (Figure 2B (Krajka et al., 2022). Similarly, a Tubb4a mutagenesis result in abnormal myelination and microtubule accumulation in oligodendrocytes (not in axon of neurons) (Duncan et al., 1992) (Figure 2A). Tau isoform with three repeats of the microtubule-binding motif (3R-Tau) has also been shown to be up-regulated or accumulated in damaged area (demyelination lesion) of stroke model mice (Villa González et al., 2020) (Figure 2C). Similarly, a FTDP-17 mutation, delK280, in the tau gene enhances the expression of 3R-Tau and also is associated with tau inclusions in oligodendrocytes (van Swieten et al., 2007).

**FIGURE 2:**
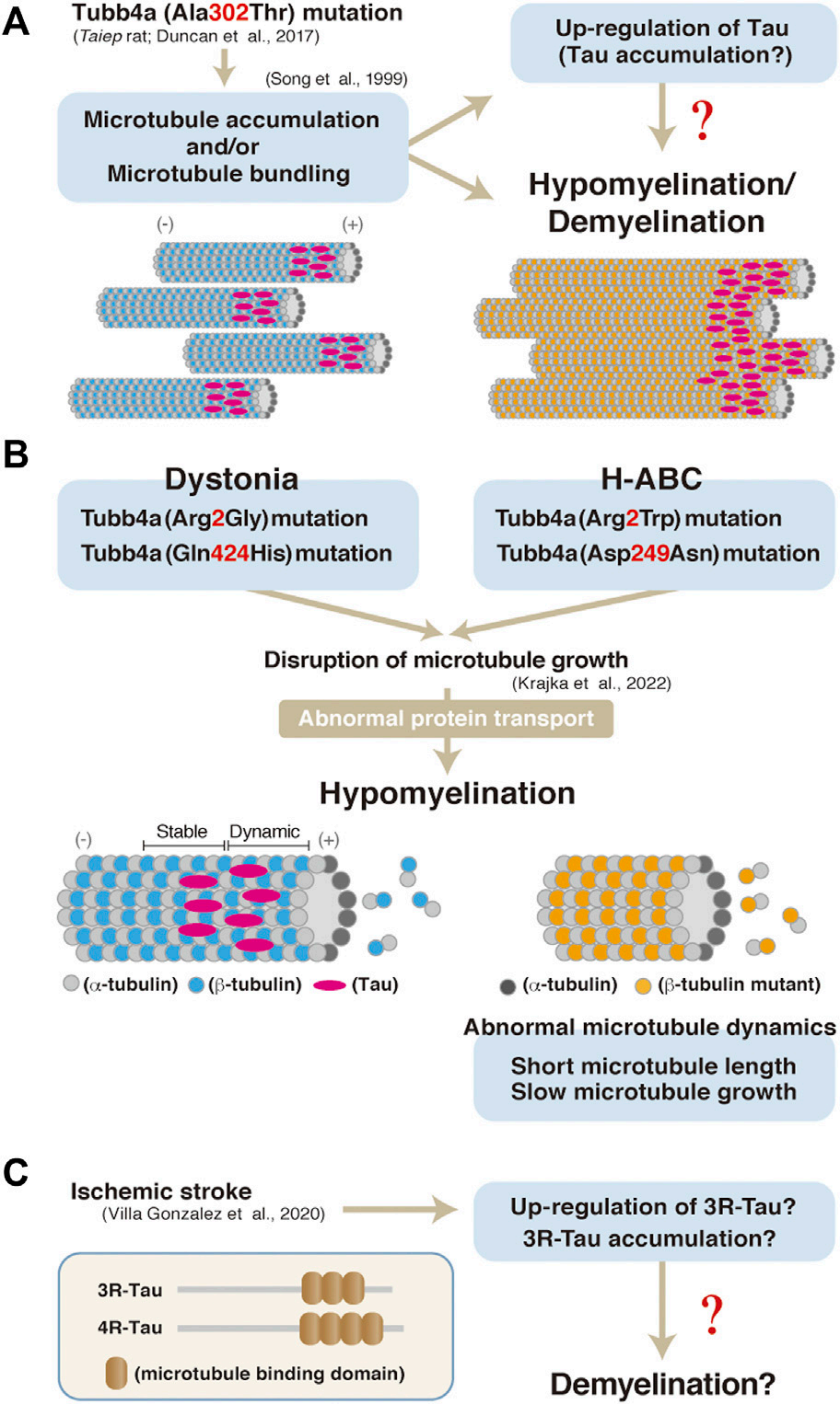
**(A)** A model of hypomyelination and/or demyelination through Tubb4a and possibly Tau in Taubb4a mutant mice. **(B)** A possibly molecular pathogenesis through Tubb4a mutation in oligodendrocytes (Krajka et al., 2022). **(C)** Up-regulation or accumulation of 3R-Tau in ischemic stroke model mice and a possible model of pathogenesis of demyelination through Tau in the mice. Representation of 3R and 4R Tau isoform structure (Villa González et al., 2020).

These findings implicate that 1) microtubule-stability and dynamics, and 2) abnormal expression, phosphorylation, and aggregation of Tau are associated with pathological dysfunction of oligodendrocytes. Tau might be needed to compensate the dysfunctions of microtubules in oligodendrocytes, or excess tau possibly induces hypomyelination and/or demyelination. Therefore, studying Tau in various models of oligodendrocyte disorders would benefit the understanding of the pathophysiology, which might identify tau as a new therapeutic target for these diseases, and also may provide insights into the physiological roles of Tau in oligodendrocytes.
